# YOLOv5‐based Detection and Classification of Early and Late Mild Cognitive Impairment through Corpus Callosum Analysis

**DOI:** 10.1002/alz.087878

**Published:** 2025-01-09

**Authors:** Vamshi Krishna Kancharla, Neelam Sinha

**Affiliations:** ^1^ Center for Brain Research(CBR), Indian Institute of Science, Bangalore, Karnataka India; ^2^ Centre for Brain Research (CBR), Indian Institute of Science, Bengaluru, Karnataka India

## Abstract

**Background:**

Alzheimer’s disease is a progressive neurodegenerative disorder that mainly affects the brain resulting gradual decline in a cognitive function, memory impairment, alterations in behavior, potentially resulting in the inability to engage in a conversation and react to the surroundings. Corpus callosum (CC) is the principal white fabric matter present in the center of the brain that connects the left and right cerebral hemispheres. Neurodegenerative diseases can impact the size and structure of the CC, leading to its atrophy and dysfunction. This study aims to detect the CC in a given Structural MRI and classify Early Mild Cognitive Impairment (EMCI) vs. Late Mild Cognitive Impairment (LMCI). This study introduces the prospect of utilizing a YOLOv5‐based framework for CC detection, aiming to distinguish between individuals with EMCI and LMCI. In addition, we have also interpreted our results using Eigen CAM.

**Method:**

In this study, we proposed a Fine‐tuned Yolov5 based object detection model for detecting CC and classifying EMCI vs LMCI. Unlike previous studies that focused solely on CC (texture analysis) for detecting MCI, our method considers both CC and the surrounding context for better EMCI vs LMCI classification. In our approach, we used MRI slices along with the corpus callosum area, enclosed in a bounding box tightly fitted to the CC coordinates. The YOLOv5 model consists of three parts: the backbone extracts features, the neck combines features at different scales, and the head makes final predictions. In this case, the object of interest for the model is the CC to classify EMCI vs LMCI.

**Result:**

The dataset used in this study was obtained from ADNI and consists of total 100 subjects, evenly distributed between EMCI vs LCMI. The dataset was partitioned into 80% for training and 20% for testing. We achieved 97% accuracy on test dataset.

**Conclusion:**

This study demonstrates YOLOv5’s efficiency in CC detection for EMCI and LMCI classification. The classification results are further interpreted using Eigen CAM. In future Fine‐tunning the model parameters and exploring other CAM varients can improve the results.

